# A pilot validation of a modified Illness Perceptions Questionnaire designed to predict response to cognitive therapy for psychosis

**DOI:** 10.1016/j.jbtep.2014.06.003

**Published:** 2014-12

**Authors:** Elena Marcus, Philippa Garety, John Weinman, Richard Emsley, Graham Dunn, Paul Bebbington, Daniel Freeman, Elizabeth Kuipers, David Fowler, Amy Hardy, Helen Waller, Suzanne Jolley

**Affiliations:** a King's College London, Institute of Psychiatry, Department of Psychology, PO77, De Crespigny Park, Denmark Hill, London SE5 8AF, UK; b King's College London, Institute of Pharmaceutical Science, 5th Floor Franklin Wilkins Building, 150 Stamford Street, London SE1 9NH, UK; c Centre for Biostatistics, Institute of Population Health, The University of Manchester, Manchester Academic Health Science Centre, 4.304 Jean McFarlane Building, Oxford Road, Manchester M13 9PL, UK; d Division of Psychiatry, University College, London, 67-73 Riding House Street, London W1W 7EJ, UK; e University of Oxford, Department of Psychiatry, Warneford Hospital, Oxford OX3 7JX, UK; f School of Psychology, University of Sussex, Pevensey Building, Falmer BN1 9QH, UK

**Keywords:** Schizophrenia, CBT, Engagement, IPQ, Therapy outcome

## Abstract

**Background and objectives:**

Clinical responsiveness to cognitive behavioural therapy for psychosis (CBTp) varies. Recent research has demonstrated that illness perceptions predict active engagement in therapy, and, thereby, better outcomes. In this study, we aimed to investigate the psychometric properties of a modification of the Illness Perceptions Questionnaire (M-IPQ) designed to predict response following CBTp.

**Methods:**

Fifty-six participants with persistent, distressing delusions completed the M-IPQ; forty before a brief CBT intervention targeting persecutory ideation and sixteen before and after a control condition. Additional predictors of outcome (delusional conviction, symptom severity and belief inflexibility) were assessed at baseline. Outcomes were assessed at baseline and at follow-up four to eight weeks later.

**Results:**

The M-IPQ comprised two factors measuring problem duration and therapy-specific perceptions of Cure/Control. Associated subscales, formed by summing the relevant items for each factor, were reliable in their structure. The Cure/Control subscale was also reliable over time; showed convergent validity with other predictors of outcome; predicted therapy outcomes; and differentially predicted treatment effects.

**Limitations:**

We measured outcome without an associated measure of engagement, in a small sample. Findings are consistent with hypothesis and existing research, but require replication in a larger, purposively recruited sample.

**Conclusions:**

The Cure/Control subscale of the M-IPQ shows promise as a predictor of response to therapy. Specifically targeting these illness perceptions in the early stages of cognitive behavioural therapy may improve engagement and, consequently, outcomes.

## Highlights

• Illness beliefs predicted response to cognitive therapy in psychosis (CBTp).
• Key predictors concerned the likelihood of curing or controlling problems.
• The Cure/control subscale was expanded to include CBT-specific change items.
• Findings could help to more efficiently target limited provision in routine services.
• ‘Pre-therapy’ interventions could target these beliefs to improve outcomes.

## 1 Introduction

Cognitive behavioural therapy for people with psychosis (CBTp) is effective in reducing symptoms, but effect sizes are modest, and individual outcomes are heterogeneous (NICE, 2009, NICE, 2014). The degree of engagement in therapy also varies: approximately half of those offered therapy in routine services will refuse (Prytys, Garety, Jolley, Onwumere, & Craig, 2011), and, even in research studies, although dropout rates may be low (Villeneuve, Potvin, Leasage, & Nicole, 2010), only a proportion of those accepting therapy are likely to engage in the full range of therapy techniques offered (Dunn et al., 2012). In the Prevention of Relapse in Psychosis trial (PRP, Garety et al., 2008), fewer than half (41%) of those allocated to the CBT arm of the trial received ‘full’ therapy (i.e. including active CBT strategies). While full therapy was associated with significant clinical improvement (increased time in remission and lower levels of psychotic and affective symptoms), those receiving a more limited range of interventions (‘partial’ therapy; primarily engagement and assessment work) showed a small deterioration, although this did not reach statistical significance (Dunn et al., 2012).

The predictors of engagement reported by Dunn et al. (2012) were primarily demographic. However, Freeman, Dunn, Garety, et al. (2013) later demonstrated that engagement in full therapy, and thereby response to CBTp, was predicted by particular beliefs held by the individual about their difficulties. Compared to those receiving partial therapy or ‘no therapy’ (i.e. non-attenders or early drop-out), those engaging in full therapy tended to endorse more internal, psychological causes of their presenting problems, expected their problems to last longer, and believed that the problem could be cured or controlled (Freeman, Dunn, Garety, et al., 2013).

These secondary appraisals of the nature, meaning and management of the problem were assessed in the PRP trial using the Illness Perceptions Questionnaire (IPQ, Weinman, Petrie, Moss-Morris, & Horne, 1996). The IPQ was originally developed to measure patients' beliefs about their physical health problems, in order to improve understanding of their responses to illness and their adherence to a range of healthcare interventions. It draws on the ‘cognitive representation’ of illness specified in Leventhal's self-regulatory model, comprising beliefs in five interrelated domains: i) the identity and nature of the illness; ii) the cause of the illness; iii) the likely duration of the illness; iv) the consequences of the illness (i.e. expected effects or impact); and v) whether and how the illness can be cured or managed. Beliefs about cure and control include appraisals of the available treatments, their likely impact, and the extent to which one's own efforts at management are likely to be effective. Following a perceived health threat, the cognitive representation of illness determines the individual's practical and emotional responses. These are manifested as adjustment and coping behaviours, and include engagement with and adherence to the recommended treatment regimen, such as medication, rehabilitation, or psychotherapy (Broadbent et al., 2006, Leventhal et al., 1984, Moss-Morris et al., 2002). The IPQ has good psychometric properties, and a growing body of research supports the influence of illness perceptions upon recovery across a broad spectrum of physical health problems (e.g. Petrie et al., 2007, Petrie and Weinman, 2006, Sirri et al., 2013, Stewart and Yuen, 2011). Moreover, in trials of psychoeducational and motivational interventions designed to promote engagement in recommended treatments, illness perceptions have been demonstrated to mediate changes in treatment adherence and outcome (e.g. Broadbent et al., 2009, Chilcot and Moss-Morris, 2013, Foster et al., 2008, Goodman et al., 2013, Juergens et al., 2010, Kucukarslan, 2012, Petrie et al., 2002, Petrie et al., 1996, Rose et al., 2012, Scharloo et al., 2010, Scharloo et al., 2007).

Similar associations of illness perceptions with adjustment, recovery and engagement with services have been reported for people with mental health problems, including psychosis (Baines and Wittkowski, 2013, Broadbent et al., 2008, Lobban et al., 2003, Lobban et al., 2004, Lobban et al., 2005, Løvvik et al., 2014, Watson et al., 2006, Williams and Steer, 2011). As in the physical health research, the focus of these studies has been engagement with recommended treatment in the broad sense of a care regimen, rather than with talking therapies specifically. However, factors shown to predict uptake of talking therapies such as expectations of change; self-efficacy; and the degree of ‘fit’ between the person's causal model and the therapy offered (e.g. Dozois et al., 2004, Gearing et al., 2014, Heins et al., 2013, Lewin et al., 2011, Trockel et al., 2014) link plausibly with the timeline, cure/control, and cause components of the IPQ that were found to predict therapy uptake in the PRP trial.

Being able to predict engagement in and response to therapy in routine care is an important goal, with the potential both to improve the efficiency of services and to facilitate the development of alternative approaches tailored to suit those who find CBTp unacceptable or unhelpful. Our aim in this study was to pilot a modification of the IPQ, designed specifically to predict response following cognitive behavioural therapy in people with psychosis. Thus, in the first part of the study we examined the psychometric properties of the measure: internal consistency, factor structure, test–retest reliability, and the extent to which the questionnaire correlated with known predictors of outcome. The second part of the study was a prospective investigation of the ability of the modified measure to: i) predict clinical outcome in participants receiving a brief cognitive behavioural intervention, and ii) to differentially predict response for an intervention group compared to a no-intervention control group.

## 2 Material and methods

### 2.1 Participants

Participants were recruited from the last cohort of the Cognitive Mechanisms of Change in Delusions study (ISRCTN 59501939), carried out by the Psychosis Research Partnership. Therapy participants completed a brief, targeted intervention for delusions, with a focus on persecutory content (Freeman et al., 2014, Waller et al., 2011). Recruitment for the current study took place over 18 months, from July 2010 to January 2012, in community adult mental health services in NHS trusts in the South East and East of England. Inclusion criteria for individual participants were: i) a current delusion, assessed using the Schedules for Clinical Assessment in Neuropsychiatry (SCAN, World Health Organization, 1992a, World Health Organization, 1992b), and rated as distressing (>0) on a visual analogue scale; ii) a diagnosis of a schizophrenia spectrum psychosis (ICD-10, F20-29, World Health Organization, 1992a, World Health Organization, 1992b) which was verified by SCAN interview; iii) aged 18–65 at study entry; and iv) a sufficient grasp of English to complete measures and participate in the intervention. In order to recruit a group with persistent, stable delusions, self-rated delusional conviction had to have consistently reached a minimum of 50%, with no major psychotic relapses or crises, over the preceding three months. Exclusion criteria were: a primary diagnosis of alcohol or drug dependency; an organic syndrome; or a learning disability.

### 2.2 Measures

Demographic and clinical data (age, gender, ethnicity and length of illness in years) were self-reported by participants and corroborated by the clinical record.

#### 2.2.1 Modified Illness Perceptions Questionnaire (M-IPQ, after Weinman et al., 1996)

The modified measure comprised 14 items (Table 2). Eleven were those found to predict engagement by Freeman and colleagues, drawn from the original IPQ (Weinman et al., 1996). Wording for these items had been modified for use with people with psychosis to ‘problems/illness’ rather than ‘illness’ to accommodate respondents who did not consider themselves to have an illness (Jolley and Garety, 2004, Watson et al., 2006). To these we added three new items, drawn from the talking therapies outcome literature, and designed to measure expectations of change and the extent to which a cognitive behavioural approach fitted with the person's own ideas about what would help with their problems. For the current study, the item ‘My treatment will be effective in curing my current problems/illness’ was changed to ‘Talking therapy will be effective in improving my current problems/illness’. Each item was rated on a five- point Likert scale in opinion format, ranging from *Strongly Disagree* to *Strongly Agree.* Higher scores represented increasing likelihood of engagement (i.e. higher levels of cure-control, greater optimism and expectation of change, longer perceived timeline, and stronger endorsement of psychological causes and the relevance of cognitive therapy ideas). The original IPQ has good psychometric properties; the psychometric properties of the modified measure were to be assessed.

#### 2.2.2 Clinical outcome

Persecutory ideation was measured using a brief scale of five visual analogue ratings, taken from Green et al.'s (2008) Paranoid Thought Scales, assessing ideas of persecution and reference. Items were selected for high loadings on their respective subscales, and to ensure coverage of a range of aspects of paranoia. Items were: I am being deliberately harmed or upset; I am being followed; There is a conspiracy against me; I am being persecuted; I am being laughed at behind my back. Each item was rated from 0 (not at all) to 100% (totally), with higher scores indicating a greater level of persecutory ideation, and the mean of all five scales forming an overall *paranoia* score. The scale has good internal reliability (Cronbach's alpha = 0.86; Freeman, Dunn, Fowler, et al., 2013; 0.82 for the current, overlapping, sample).

#### 2.2.3 Predictors of outcome

Greater symptom severity at baseline, higher levels of conviction, and belief inflexibility, although not specifically demonstrated to moderate treatment effects, have all been associated with poorer outcomes in trials of CBTp (Brabban et al., 2009, Garety et al., 1997, Naeem et al., 2008). For the current study, participants provided personalised ratings of their degree of conviction in their delusion, indicating how much they believed it from 0 (not at all) to 100 (totally). Belief inflexibility (Garety et al., 2005, So et al., 2012) was measured by reverse scoring ratings of the extent to which participants considered it possible they were mistaken in their delusion, rated from 0 (not at all) −100% (totally) (Waller et al., 2011).

### 2.3 Procedure

Participants in the current study completed the M-IPQ and other measures as part of a larger, repeated, assessment battery designed to evaluate change during brief cognitive behavioural interventions. They were participating in one of two randomised controlled trials (RCTs, randomising participants to brief intervention or a no-therapy control condition in a 2:1 or 1:1 ratio, depending on the trial) or in a case series. All the measures for the current study were completed at baseline by all participants. Control participants completed the M-IPQ at baseline (Time 1) and at a second time point two to eight weeks later (Time 2). Intervention participants completed the clinical outcome measure (paranoia scale) at baseline and at follow-up, four to eight weeks later. Measures were administered by trained research workers. Participants provided informed consent under protocols approved by the appropriate ethics and local research and development committees (London Wandsworth Research Ethics Committee 07/H0803/140), and received recompense for their time spent taking part in the study.

### 2.4 Analyses

Data were analysed using SPSS version 20 (IBM, 2011). The association of M-IPQ scores with baseline demographic and clinical characteristics (age, gender, ethnicity, length of illness) was examined in the full sample using correlational analysis and one-way ANOVA. Baseline M-IPQ scores for the full sample were subjected to a principal components analysis with an oblique rotation (direct oblimin), as the factors were not expected to be fully orthogonal. Subscales were formed by summing the items loading on each factor. Inter-item correlations and internal consistency (Cronbach's *α*) were calculated for the full scale and for each subscale. Test–retest reliability was calculated by comparing baseline to follow-up scores for control participants only, using intraclass correlations. The relationship of the M-IPQ to other known predictors of outcome (baseline symptom severity, belief inflexibility, and conviction) was assessed in the full sample by Pearson correlations. Associations of the M-IPQ with clinical outcome following therapy, were assessed for the therapy group only, using linear regression analysis, with paranoia score at follow-up as the dependent variable, and the M-IPQ subscales as predictors, controlling for baseline paranoia in the first block, and, in the second block, for other predictors of outcome, in order to assess the ability of the M-IPQ to predict outcome over and above known predictors (i.e. those identified by previous literature). The ability of the M-IPQ to predict response to therapy was assessed in a second series of regression analyses including all participants, both control and therapy. A separate analysis was conducted for each M-IPQ subscale associated with outcome. Paranoia score at follow-up was the dependent variable in each analysis; the key independent variable was an interaction term of the selected M-IPQ subscale × therapy allocation. Analyses controlled for baseline paranoia, the other three outcome predictors, the selected M-IPQ subscale and therapy allocation. A backwards selection procedure was employed. The analysis was repeated, excluding the case series participants, as these had not been randomly allocated to treatment condition. There was a single missing item for one participant on the M-IPQ, which was replaced by the mean (of the remaining items) for the factor analysis. Participants with missing data were otherwise excluded and sample sizes reported.

## 3 Results

### 3.1 Participants

A total of 56 people with schizophrenia spectrum psychosis completed the M-IPQ and therefore participated in the study. All participants had a current, stable, distressing and strongly held delusion. Forty participants completed the brief intervention, either as part of a larger RCT (*n* = 34) or a case series (*n* = 6). Sixteen participants were in the no-therapy control group. The sample was mainly male, and of mixed ethnicity, with schizophrenia being the most common diagnosis. Demographic and clinical characteristics are shown in Table 1.

**Table 1:** Demographic and clinical characteristics.

Variable	Total (*n* = 56)	Brief therapy (*n* = 40)
	Mean (SD)	
Age (years)	42.4 (10.8)	41.0 (10.7)
Length of illness (years)	15.3 (10.3)	13.5 (10.1)
Baseline paranoia	36.4 (28.8)	31.1 (24.3)
Follow-up paranoia	29.3 (27.8, *n* = 54)	25.9 (27.4, *n* = 38)
Baseline outcome predictors conviction	79.5 (26.8, *n* = 55)	78.0 (28.3, *n* = 39)
Belief inflexibility	77.7 (27.8, *n* = 53)	76.8 (28.4, *n* = 38)
Delusion distress	65.4 (32.6, *n* = 55)	58.2 (22.3, *n* = 39)

*Gender (n)*		
Male	38	28
Female	18	12

*Ethnicity (n)*		
White-British/Irish/other	30	23
Black-Caribbean/African/mixed/other	22	16
Asian-mixed/other	4	1

*Diagnosis (n)*	*(n = 54)*	*(n = 39)*
Schizophrenia	48	35
Paranoid schizophrenia	1	1
Schizoaffective disorder	3	3
Delusional disorder	1	0
Other non-organic psychotic disorder	1	0

### 3.2 Reliability and factor structure of the M-IPQ

The internal consistency of the full M-IPQ scale was just acceptable (Cronbach's *α* = 0.7), with some collinearity amongst the items, and multiple inter-item correlations of 0.3 and above, indicating that the derivation of factors was appropriate. Principal components analysis, with direct oblimin rotation, resulted in the extraction of three related components with eigenvalues > 1; the first three principal components, respectively accounted for 39%, 12% and 11% of the variance in M-IPQ scores (62% of the variance in total).

Two of the three reverse scored items, which were drawn from the original IPQ (Items 5 and 7), and were designed to tap hopelessness and a fatalistic approach to change, did not appear to correlate as expected. The analysis was repeated with these items omitted, which reproduced the same structure, with very slightly altered item loadings. The three components and both sets of item loadings are shown in Table 2, and were labelled Cure/Control+, Timeline and Internal/External Causality.

**Table 2:** Item loadings for each factor of the modified Illness Perceptions Questionnaire (M-IPQ, *n* = 56).

Item	Factor 1 Cure/control	Factor 2 Timeline	Factor 3 Causes
1. My problems can improve^a^	**0.8**		
2. There is a lot which I can do to improve my problems	**0.9**		
3. What I do can determine whether my current problems/illness get better or worse	**0.7 (0.8)**		
4. My current problems/illness will improve in time	**0.7**	0.3	
5. There is very little that can be done to improve my current problems (R)	0.4		**0.5**
6. Talking therapy will be effective in improving my current problems/illness^b^	**0.6 (0.7)**		
7. Recovery from my current problems is largely dependent on fate or chance (R)			**0.8**
8. My current problems/illness will last a short time (R)		**0.7**	
9. My current problems/illness are likely to be permanent rather than temporary		**0.8 (0.9)**	
10. My current problems/illness will last for a long time		**0.8**	
11. My state of mind played a major part in causing my current problems/illness	0.6		**−0.5 (0.4)**
12. Something about my personality played a role in causing my current problems/illness			**−0.5 (0.9)**
13. Changing the way I think or the way I do things can improve my problems^a^	**0.8**		
14. Looking at things differently can be helpful^a^	**0.8**		

Key: coefficients < 0.3 are suppressed; bold text denotes item considered part of this factor; () = coefficient if different once items 5 and 7 are removed; if not noted, coefficients remain constant.

a New items assessing expectation of change (Item 1) and Cognitive model fit (Items 13 and 14).

b Item wording modified to ‘Talking therapy’ rather than ‘Treatment’.

M-IPQ subscale scores were calculated by simply adding the relevant items (allowing for sign reversals, where relevant). The Cure/Control+ subscale comprised seven items (Items 1, 2, 3, 4, 6, 13, 14) which assessed the extent to which the individual perceived themselves to be able to control and change their current problems (e.g. ‘*There is a lot which I can do to improve my problems*’). The new items assessing expectations of change, and those assessing CBT fit loaded on the Cure/Control factor, and the subscale showed good internal reliability (Cronbach's *α* = 0.9) and high inter-item correlations, suggesting that it is tapping a broader Cure/Control construct, incorporating specific beliefs about therapy. The Timeline subscale comprised three items from the original IPQ (Items 8, 9, 10) which assessed the individual's perceived duration of their problems/illness (e.g. ‘*My current problems/illness will last a long time*’). Internal reliability was just acceptable (Cronbach's *α* = 0.7). Two items (Items 11 and 12) which measured the extent to which the person considered their problems/illness to be internally caused (e.g. ‘*My state of mind played a major part in causing my current problems/illness*’) loaded on the Internal/External Causality factor, together with the two reverse scored items (Items 5 and 7) designed to access hopelessness and fatalism about change (in contrast to self-efficacy). Internal reliability across these items was unacceptably low, and all inter-item correlations were below 0.2. For subsequent analyses, therefore, items 5 and 7 were omitted, and the two internal causality items were examined individually. Inter-item correlations are shown in Table 3; M-IPQ subscale mean scores are given in Table 4.

**Table 3:** Inter-item correlations for the modified Illness Perceptions Questionnaire (M-IPQ, *n* = 56).

	Item	1	2	3	4	6	13	14	8	9	10	11	12	5	7
Cure/control+	1	–	0.7	0.5	0.6	0.3	0.5	0.6				0.4		0.4	
	2	0.7	–	0.5	0.7	0.5	0.7	0.7			−0.3	0.4		0.4	
	3	0.5	0.5	–	0.6	0.5	0.5	0.5	−0.3			0.3			
	4	0.6	0.7	0.6	–	0.5	0.6	0.7	−0.5		−0.4	0.3		0.3	
	6	0.3	0.5	0.5	0.5	–	0.5	0.6	−0.4		−0.3	0.3		0.3	
	13	0.5	0.7	0.5	0.6	0.5	–	0.6				0.4		0.3	
	14	0.6	0.7	0.5	0.7	0.6	0.6	–	−0.3		−0.4	0.4		0.4	
Timeline	8			−0.3	−0.5	−0.4		−0.3	–	0.3	0.5	−0.3			
	9				−0.3				0.3	–	0.6				
	10		−0.3		−0.4	−0.3		−0.4	0.5	0.6	–				
Internal/external cause	11	0.4	0.4	0.3	0.3	0.3	0.4	0.4	−0.3			–			−0.3
	12												–		
	5	0.4	0.4		0.3	0.3	0.3	0.4						–	0.3
	7											−0.3		0.3	–

Correlations < 0.3 are suppressed.

**Table 4:** Mean modified Illness Perceptions Questionnaire (M-IPQ) subscale scores at baseline and test–retest, with regression results showing the prediction of clinical outcomes following therapy for each subscale.

Subscale/item	Total baseline (*n* = 56)	Control T1 (*n* = 15^a^)	Control T2 (*n* = 15^a^)	Therapy baseline (*n* = 40)	Prediction of outcome (*n* = 34^b^)
	Mean (SD)				*β* (*p*)^c^
Cure/control+	26.1 (6.2)	25.6 (6.0)	24.3 (6.2)	26.1 (6.3)	−0.4 (0.03)
Timeline	10.1 (2.9)	11.5 (2.3)	11.6 (2.4)	9.7 (3.0)	−0.3 (0.1)
State of mind	3.5 (1.3)	3.9 (1.1)	3.9 (1.0)	3.3 (1.4)	0.2 (0.2)
Personality	3.3 (1.2)	3.3 (1.1)	3.4 (1.0)	3.3 (1.3)	−0.1 (0.4)

a One control participant did not complete post measures.

b Therapy group only, 6 participants with missing data on one or more variables.

c *F*(8,26) = 3.3, *p* = 0.01; *r* = 0.7, *R*^2^ = 0.35; controlling for baseline paranoia and then for other predictors of outcome (baseline conviction, belief inflexibility and distress).

### 3.3 Test–retest reliability

Test–retest reliability was calculated for the control group participants only and was good for the Cure/Control+ subscale (*r* = 0.9, *p* < 0.001, *n* = 15), but questionable for the Timeline subscale (*r* = 0.6, *p* < 0.001, *n* = 15). The internal causality items showed an unacceptable test–retest reliability (State of Mind: *r* = 0.1, *p* = 0.5, *n* = 15; Personality: *r* = 0.4, *p* = 0.01, *n* = 15). Mean M-IPQ subscale scores at Time 1 and Time 2 are shown in Table 4.

### 3.4 Demographic and clinical characteristics and the M-IPQ

There were few significant associations between M-IPQ scores and demographic or clinical characteristics. The M-IPQ was unrelated to age, or recruitment centre, but Cure/Control+ scores were associated with length of illness (*r* = 0.3, *p* = 0.03), such that a longer duration of illness was associated with greater perceptions of control, and the Personality item was associated with Ethnicity (*F*(2,53) = 8.8, *p* < 0.001) and Gender (*F*(1,54) = 4.5, *p* < 0.05), such that male participants and those identifying themselves as from a White ethnic group were more likely to endorse internal causes. No other associations with demographic variables reached significance (*r* values < 0.25, *p* values > 0.05; *F* values all < 3, *p* values > 0.05).

### 3.5 Convergent validity

Baseline symptom severity and belief inflexibility scores are shown in Table 1. Associations of predictors of outcome with demographic variables were examined; the only association that was also found for the M-IPQ was with gender, such that male participants were more likely to be flexible in their beliefs (*F*(1,51) = 6.1, *p* = 0.02). The Cure/Control+ subscale was correlated significantly or at a trend level with all other outcome predictors except baseline paranoia (distress *r* = −0.3, *p* < 0.05; conviction: *r* = −0.3, *p* = 0.06; belief inflexibility: *r* = −0.3, *p* = 0.02). Of the other M-IPQ components, only Personality showed a trend association with conviction (*r* = 0.2, *p* = 0.07); otherwise associations were insignificant (*r* values < 0.2, *p* values > 0.2). Controlling for gender did not alter the lack of association between belief inflexibility and Personality.

### 3.6 Prediction of clinical change scores following therapy

Mean paranoia scores at baseline and follow-up for the therapy group are shown in Table 1. Therapy outcomes were not significantly related to demographic variables (*F* values < 3; *r* values ≤ 0.2, *p* values > 0.05). Linear regression analysis showed significant associations of the Cure/Control+ subscale at baseline with paranoia outcomes following therapy, controlling for baseline paranoia. The overall model was significant and accounted for 35% of the variance in clinical outcome. Cure/Control+ remained a significant predictor irrespective of controlling for other predictors of outcome (baseline distress, conviction and belief inflexibility; Table 4). Only Cure/Control+ of the M-IPQ subscales was therefore entered into the second series of regression analyses, including all participants. The final model accounted for 46% of the variance in paranoia outcomes at follow-up, with a significant contribution from the Cure/Control × therapy allocation interaction (*β* = 1.0, *p* = 0.04), controlling for allocation (*β* = −1.0, *p* = 0.04), Cure/Control+ (*β* = −0.6, *p* = 0.004), and baseline paranoia (*β* = 0.6, *p* < 0.001). Excluding the case series participants resulted in an almost identical model (45% of the variance accounted for; Cure/Control × therapy allocation interaction (*β* = 1.2, *p* = 0.03), controlling for allocation (*β* = −1.0, *p* = 0.04), Cure/Control+ (*β* = −0.6, *p* = 0.004), and baseline paranoia (*β* = 0.6, *p* < 0.001)).

## 4 Discussion

We conducted a pilot validation of a modified version of the Illness Perceptions Questionnaire (M-IPQ), designed to predict response following cognitive behavioural therapy for people with psychosis (CBTp). We shortened the IPQ to include only those items predicting therapy uptake (Freeman, Dunn, Garety, et al., 2013), and added three new items assessing general therapy engagement. The measure showed acceptable psychometric properties, and formed three factors, from which two reliable subscales were derived. The first subscale represented an expanded Cure/Control construct, incorporating new items measuring hopefulness, self-efficacy, and therapy fit (‘Cure/Control+’). The second subscale assessed perceptions of the duration of the problem (Timeline). The Cure/Control+ subscale was associated with established predictors of outcome, and itself predicted response to therapy over time.

The factor structure of the modified measure partially replicated that of the original IPQ. The added items, expectation of change and therapy fit, did not form separate components, but instead loaded onto an expanded Cure/Control construct, which had very high internal and test–retest reliability. Our findings may indicate that the Cure/Control concept should be expanded to include ‘hope’ and ‘fit’, which both feature prominently in the literature on engagement and change in psychological therapy and physical health interventions, and have been demonstrated to predict outcome in CBT (Gearing et al., 2014; Sirri et al., 2013). A clear Timeline factor was found, replicating previous findings. The Timeline subscale had good internal reliability, but questionable reliability over time. Internal causes formed a weak factor, loading with the reverse scored Cure/Control items assessing a fatalistic attitude to recovery, and lack of expectation of change. Both internal and test–retest reliability were poor. The reverse scored items were excluded from the final measure, as both the inter-item correlations and factor loadings suggested that the meaning and the direction of the items were misinterpreted by participants. The poor reliability of the items assessing causes is well-documented, being attributed to the high potential for idiosyncratic interpretations of the items, resulting in their exclusion from analyses, or inclusion as separate items (Lobban et al., 2004, Watson et al., 2006). However, in common with other studies of illness perceptions in mental health (Broadbent et al., 2008, Freeman et al., 2013), we found a relationship between attribution to internal causes and Cure/Control, with the State of Mind item loading on the expanded Cure/Control factor, as well as the weaker Causes factor. The association suggests that internal attributions of cause may be treated as a component of therapy fit. In this context, re-wording the item may be useful to ensure it unambiguously captures the therapy-relevant notion of change in oneself being both possible and helpful.

The Cure/Control factor showed moderate construct validity, evidencing small but consistent correlations with measures of symptom severity, conviction, and belief flexibility, which have all been demonstrated to predict outcome in previous research. Perceptions of cure control were associated with longer duration of illness, possibly reflecting increasing ability to cope with a stable presentation over time. We also found that higher perceived levels of Cure/Control were associated with therapy outcomes. Moreover, the significant association of the Cure/Control+ × therapy allocation interaction term with outcomes indicates that the subscale predicts outcome differentially for the brief CBTp and control groups. This is important, and supports the future use of the measure as a potential predictor of response to therapy. However, neither causal attributions nor timeline predicted outcome in our sample, contrasting with previous studies (e.g. Freeman, Dunn, Garety, et al., 2013). The differences may be attributable to characteristics of the presenting problems (e.g. stable or relapsing; early or longer term) and the intensity and duration of the intervention, affecting motivation to engage. Systematic investigation of the influence of these variables on the prediction of engagement and outcome would be a useful focus of future study.

Participants completed the measure without undue difficulty, and their responses were coherent and meaningful. The findings provide further evidence of the clinical utility and feasibility of measuring illness perceptions in people with psychosis, and, with small modifications, the suitability of the IPQ for this group, both of which have previously been questioned (Kinderman, Setzu, Lobban, & Salmon, 2006). The high inter-item correlations support shortening the measure, and this would increase its utility for routine assessment.

### 4.1 Limitations

As participants were recruited for a specific research project, with a particular clinical presentation, and an ethnically diverse and urban demographic, the generalisability of findings may be limited. Future research should aim to clarify the variations in predictors of engagement and outcome for specific groups. Secondly, although response to therapy was measured, engagement was not, so we can only infer, by association with positive outcome, the extent to which participants engaged with particular intervention strategies. The ability of the M-IPQ to predict engagement independently of outcome therefore remains untested in this study. Thirdly, the M-IPQ comprised those items from the original version of the IPQ that were associated with engagement in the PRP trial. Content could be improved by drawing on the subsequent modifications to the IPQ. Fourthly, the M-IPQ and the primary outcome measure are self-reported. Although M-IPQ items are carefully phrased to sidestep issues of acknowledging illness, ratings rely on the person's own perception of their difficulties and are potentially influenced by poor insight. Finally, as a small pilot validation, the study is underpowered for a full factor analysis, and for the multiple associations tested. The control group is particularly small, and allocation to group was not fully randomised. The findings regarding prediction of response to therapy should therefore be treated as preliminary. Nevertheless, findings are consistent with the existing literature and with hypothesised associations.

### 4.2 Implications

This is the first study prospectively validating the modified IPQ as a predictor of treatment outcome in CBT for psychosis. Our findings suggest that perceptions of control and optimism about change increase the likelihood of a positive outcome following therapy. Previous research has shown that perceptions of control also increase the likelihood of engagement in the full range of therapy techniques (Freeman, Dunn, Garety, et al., 2013), which in turn mediates positive outcomes (Dunn et al., 2012). In physical health research, brief interventions focused on modifying illness perceptions have been successful in increasing engagement in treatment. It is possible, therefore, that engagement in CBTp could be increased by a focus on illness perceptions in the early stages of therapy. Specifically, modifying illness perceptions to increase service users' perceptions of the controllability of their difficulties and their expectation of change ought to increase engagement, with the potential to improve outcomes, and reduce later dropout. Illness perceptions frameworks have also been used as a means of training the wider healthcare workforce to communicate in a more effective, individually tailored and patient-centred manner about interventions for a range of physical health problems, with positive impact on outcomes (e.g. Glattacker, Heyduck, & Meffert, 2012). As clinical communication is a known area of particular difficulty in the treatment of people with psychosis (e.g. McCabe et al., 2013), a similar approach could inform workforce development initiatives to improve treatment adherence and promote recovery from psychosis.

### 4.3 Conclusions

Our pilot validation demonstrated the modified version of the Illness Perceptions Questionnaire (M-IPQ) to have good psychometric properties. An expanded Cure/Control subscale, incorporating expectations of change and degree of fit between a CBTp framework and the service user's own appraisals, was associated with existing outcome predictors, and predicted outcomes following therapy. The measure has potential for use as a screening tool in routine services. Brief interventions specifically targeting perceptions of the controllability of the problem, and the possibility of change may increase active engagement in CBTp, thereby increasing the likelihood of a successful outcome.

## Financial support

The PRP group work was supported by a project grant (grant number 085396) from the Wellcome Trust. PG and EK were part funded by the Department of Health via the National Institute for Health Research (NIHR) Specialist Biomedical Research Centre for Mental Health award to South London and Maudsley NHS Foundation Trust (SLaM) and the Institute of Psychiatry at King's College, London. DFr is supported by a Medical Research Council (MRC) Senior Clinical Fellowship.

## Ethical standards

The authors assert that all procedures contributing to this work comply with the ethical standards of the relevant national and institutional committees on human experimentation and with the Helsinki Declaration of 1975, as revised in 2008.
